# Powerpenia Should be Considered a Biomarker of Healthy Aging

**DOI:** 10.1186/s40798-024-00689-6

**Published:** 2024-03-25

**Authors:** Sandro R. Freitas, Carlos Cruz-Montecinos, Sébastien Ratel, Ronei S. Pinto

**Affiliations:** 1 Laboratório de Função Neuromuscular, Faculdade de Motricidade Humana, Universidade de Lisboa, Cruz-Quebrada, Portugal; 2 Department of Physical Therapy, Faculty of Medicine, University of Chile, Santiago, Chile; 3 Section of Research, Innovation and Development in Kinesiology, Kinesiology Unit, San José Hospital, Santiago, Chile; 4 AME2P, Clermont-Auvergne University, Clermont-Ferrand, 3533 EA France; 5 School of Physical Education, Physiotherapy and Dance, Universidade Federal do Rio Grande do Sul, Porto Alegre, RS Brazil

**Keywords:** Dynapenia, Health, Neuromuscular function, Quality of life, Sarcopenia, Sedentarism

## Abstract

To identify biomarkers that precede the decline of human function and independence during the lifespan, two important concepts have been introduced in recent decades: sarcopenia and dynapenia. While the former is originally focused on skeletal muscle loss, the latter is on maximal strength loss. Although the dynapenia concept implies the inclusion of skeletal muscle power, in practical terms, this has not been specifically addressed. For instance, only 2 out of 220 studies published between 2008 and 2023 have directly measured muscle power to classify individuals with dynapenia. As previous studies have shown a greater relevance of skeletal muscle power in healthy aging, we hereby propose the introduction of the term “powerpenia” to specifically reflect the loss of skeletal muscle power along lifespan, but also with disease and/or physical inactivity. Together with sarcopenia and dynapenia, we contend that powerpenia should be considered a biomarker of healthy aging.

**Supplementary Information:**

The online version contains supplementary material available at 10.1186/s40798-024-00689-6.

## Key Points

• Skeletal muscle power is more influential than muscle strength and mass in enhancing physical function and reducing falls in older adults, and is a more sensitive marker of disease presence and/or physical inactivity;

• The existing concepts of sarcopenia and dynapenia are commonly accepted and used among professionals and researchers in the fields of health and physical exercise. However, concept of dynapenia has been focused on skeletal muscle maximal strength rather than power, and sarcopenia does not include muscle power as a criterion.

• We propose the introduction of the term *powerpenia* to specifically address the loss of skeletal muscle power with aging, disease, and/or physical inactivity.

**Supplementary Information:**

The online version contains supplementary material available at 10.1186/s40798-024-00689-6.

## Introduction

In normal circumstances, humans seek to be physically independent in their daily living to safeguard their quality of life. However, humans are becoming increasingly sedentary in all stages of life, and this negatively impacts human skeletal muscle force-velocity properties, functional abilities, quality of life, and longevity [1–3]. Researchers and reputable advisory institutions worldwide (e.g. the World Health Organization) are now interested in promoting healthy living with a particular interest in attenuating the decline in human function, where physical exercise plays a key role.

Over the last four decades, two main biomarkers have been introduced to identify the early decline in human physical function in the later stages of life: sarcopenia and dynapenia. In 1988, Irwin H. Rosenberg proposed two terms to reflect the concept of skeletal muscle mass loss with age, specifically sarcomalacia and sarcopenia [4]. The latter term has prevailed in the community worldwide and it has been considered a fundamental criterion of human health in different stages of life and in a range of clinical conditions [5]. In 2008, Brian C. Clark and Todd M. Manini proposed the concept of dynapenia to specifically focus on the loss of skeletal muscle strength and power due to its potentially greater relevance when explaining human function decline and its poor association with muscle mass loss [6]. Some years later [7, 8], a consensus-based review of the sarcopenia concept introduced the criterion of muscle function (i.e., maximal strength production capacity used for handgrip and walking locomotion). Notably, skeletal muscle power (see definition in the next section, i.e. Powerpenia) was not included in both the original and revised sarcopenia definitions [4, 7, 9]. However, in practical terms, sarcopenia has been focused on a loss of skeletal muscle mass rather than function [10]. Thus, some researchers are currently of the opinion that sarcopenia should focus on its original concept of loss of skeletal muscle mass [11].

Over the years, a considerable number of studies have differentiated the concepts of dynapenia and sarcopenia due to three main reasons. Firstly, the onset of skeletal muscle strength loss with age is likely to occur before the onset of muscle mass loss [9, 12]. Secondly, the decrease rate is higher for muscle strength compared to mass [9, 13]. Thirdly, strength loss related to aging cannot be explained entirely by a decrease in muscle size [14]. As such, the application of terms that fit the meaning of the original concepts is justified, considering how *sarco-* means “muscle” [4], *dyna-* refers to “power, strength, or force” [6], and *penia-* means “poverty”. It should be noted that the use of the conjunction “or” between strength and power in the dynapenia original concept suggests that these functional parameters are equivalent. However, this is not the case. Human strength is defined as the force a muscle can produce to overcome a resistance while power is defined as the amount of work performed per unit of time, which can be determined by the product of muscle force and velocity [15, 16]. For instance, individuals may have similar strength capacities but with different power outcomes. As such, the assessment of both parameters requires different approaches. The rate of power loss during aging is also higher than strength [17], which means the power loss with age cannot be fully explained by loss of strength [3]. The relevance of these functional parameters during aging is also not similar, as higher association with human physical function and falls reduction has been observed for skeletal muscle power rather than strength [2]. The distinction between strength and power is also required because there are specific factors that regulate a fast voluntary muscle action [18], even if the other factors underpinning the production of both maximal strength and power are similar. This means that to classify individuals with dynapenia according to the original concept, there would have to exist a criterion regulated simultaneously by both parameters. However, this is not the case, as the criteria for diagnosing dynapenia have mainly been assessed based on maximal strength. As shown in Table 1, of the studies published between 2008 and 2023 that objectively classified individuals as having dynapenia (*n* = 220, see list in supplementary file_1), only two studies (i.e. 0.9%) used an objective measure of muscle power. Most studies (i.e. 91.8%) assessed dynapenia through the quantification of handgrip maximal strength. This discrepancy calls for the need to differentiate loss of muscle strength and loss of muscle power within the dynapenia concept, or to redefine the concept. Interestingly, in 2011, Morley et al. proposed the term *kratopenia* to characterize the “loss of force” and *dynapenia* to characterize the “loss of power” [19], although there was no subsequent adherence to this proposal. However, a clear differentiation between strength and power is warranted.

**Table 1:** Type of tests used to assess dynapenia in humans, reported in studies published between 2008 and 2023 (*n* = 220, up to August 7th) searched for in the PubMed search engine with the word “dynapenia”

Type of test to diagnose dynapenia	n	%
Handgrip	170	77.3
Handgrip and 6-m walk	20	9.1
Handgrip and isometric leg extension	5	2.3
Isometric leg extension	4	1.8
Isokinetic leg extension	4	1.8
Handgrip and sit to stand	4	1.8
Sit to stand	2	0.9
Mid-thigh pull	2	0.9
Sit to stand power	1	0.5
Phase angle (Bioelectrical impedance analysis)	1	0.5
Leg press and bench press	1	0.5
Isometric leg extension strength and power	1	0.5
Handgrip, 6-m walk and Timed Up and Go	1	0.5
Handgrip and Short Physical Performance Battery	1	0.5
Handgrip and inverted grid-hang test	1	0.5
Eccentric strength of the hip-flexors and hip abductors	1	0.5
Bench press and knee extension	1	0.5
**Inclusion of specific power measures?**	**n**	**%**
Yes	2	0.9
No	218	99.1

## Powerpenia

We hereby introduce the term *powerpenia* to specifically address the loss of skeletal muscle power induced by aging, clinical conditions, and/or physical inactivity. Consequently, we suggest that dynapenia should be focused only on skeletal muscle maximal strength. The need to identify a specific term that addresses the loss of skeletal muscle power with age has also been raised recently [20]. Notably, the term powerpenia has not been used before in the literature. We contend that the use of the prefix power- will intuitively lead individuals to the meaning of the concept, and thus justifies the proposal of a neologism term [21]. The term power has only been considered as a medical subject heading in PubMed in terms of a psychological dimension, suggesting that skeletal muscle power is not a topic considered in biomedical and health-related contexts, contrary to muscle strength (but not dynapenia) and sarcopenia. We contend that introducing the powerpenia concept will help to distinguish between muscle strength and power and intuitively convey the power decline due to aging to (non-)scientific communities [22]. With the proposal of the powerpenia concept, dynapenia should be redefined to focus solely on skeletal muscle strength. Otherwise, the testing type and criteria to diagnose dynapenia in individuals would need to be revised to be coherent with its original definition. We assume that this is much more challenging, as it would be obligatory to create double assessment and double conjugated criteria for strength and power (which do not currently exist). As mentioned previously, the original concept of dynapenia presupposed that force and power are equivalent, and in turn regulated by the same factors. While some common physiological factors may underlie both muscular strength and power (e.g. skeletal muscle mass properties), it should be noted that the specific (and different) factors underpinning skeletal muscle power and strength production capacity have been widely reported in the scientific literature [2, 12, 23]. For instance, some factors can only be attributed to power tasks due to its supraspinal dependence [18], and maximal strength poorly explains the very early rate of force development (i.e. a power-related variable) [24]. By accepting both the introduction of the powerpenia and the revised dynapenia concepts, we contend that much more specific research and discussion will be obtained, as a given doctrine only develops in the presence of solid concepts. Also, more objective and effective intervention in clinical settings for diagnosing deficits in both skeletal strength and power as well as specific training to address these deficits would be obtained. For instance, higher attention would be given to power training in aged individuals (and perhaps in some patients), as this mode of training appears to have greater influence on human physical function improvement and fall prevention [2, 13]. Despite these arguments, we admit that some criticism may be raised against the introduction of the powerpenia concept. For instance, one may argue that powerpenia could cause some confusion to existing and accepted terminology of sarcopenia and dynapenia in research and clinical practice. However, non-acceptance of the term powerpenia would require a revision of the sarcopenia testing and criteria (through the introduction of power assessment) and dynapenia (through the differentiation between strength and power, and the introduction of double testing and criteria). We contend the latter option would be much more challenging. It could also be questioned whether a differentiation between dynapenia and powerpenia would lead to improved patient outcomes with treatment strategies or preventive approaches. As the physiological factors involved in strength and power are not completely similar, we assume that much more specific interventions would be designed and thus better outcomes would be obtained.

The acceptance of the powerpenia concept would also raise awareness about the importance of skeletal muscle power in human health, as well as promote powerpenia-modifying interventions. Skeletal muscle power has been considered a better predictor of functional performance in older adults than muscle strength [2, 25], and it appears to be more important in preventing falls among older adults [26]. It is also associated with the health and function of several body structures, such as bone strength [27]. In addition, skeletal muscle power may indicate cognitive status [28], and serve as an indicator of the individual’s motivation [18, 29] and will to live [29]. Thus, specific intervention approaches would also be designed to overcome skeletal muscle power deficits if the concept is introduced. In terms of exercise prescription, a higher emphasis would be given to muscle contraction velocity during resistance training exercises. Notably, this type of exercise modality known as power training has been shown to provide better improvements in skeletal muscle power outcomes than traditional (i.e. low-velocity) strength training [30–32] However, the effects of the power exercises appear to be task-dependent, with greater adaptations in power-based motor tasks (e.g., fast walking velocity and the five sit-to-stand test), rather than in functional tasks with endurance component (e.g., six-minute walking test) [32]. Finally, the promotion of the powerpenia concept could also help to reduce the reluctance that prevails in the scientific community regarding the safety and effectiveness of power training interventions in older adults. This reluctance persists despite convincing literature having shown that skeletal muscle power capacity can be effectively improved with minimal risk among older adults, and this can also be associated with very relevant positive effects on human function and health among this population [13, 32].

Existing evidence also suggests that skeletal muscle power reduces with a greater magnitude compared to strength in the presence of certain clinical conditions, but also with increased physical inactivity [33–36]. For instance, greater deficit of lower limb power compared to strength has been found in patients with sarcopenia (i.e. 23% vs. 11%, respectively) [33], Parkinson’s disease (22% vs. 16%) [34], and type 2 diabetes (19% vs. 14%) [35]. Levels of physical (in)activity and sedentary behavior have also been associated with lower limb power in older adults, with a stronger association than strength [36]. Together, this suggests that power deficit is a much more sensitive marker in the presence of disease and/or physical inactivity than strength (and also mass) to detect skeletal muscle impairment. This justifies why powerpenia should be considered as a biomarker within clinical and health settings.

The methodological approach to measure and classify individuals with powerpenia is complex, yet to be determined, and requires future investigation. There is a high diversity of power tests that mobilize different body regions (i.e. upper vs. lower limbs) and with different levels of motor demand and complexity (e.g. horizontal jump vs. sit-to-stand tests). We acknowledge that the selection of power tests to assess the individual’s powerpenia should depend on the individual’s age group, functional status, and physical limitations. This fact is also justified by the ceiling effect that some tests may have in certain populations. For example, high performance may be obtained on the sit-to-stand test in the majority of the young adult population (or even physically active older adults), making the test irrelevant for this age group. In this case, another type of test may be more suitable as a health indicator (e.g. vertical jump). Similarly, while young individuals may be able to perform a bilateral vertical jump without difficulty, older adults with an advanced age may be unable to do this task. In these situations where individuals are unable to perform the physical task, less demanding and complex tests should be selected to identify muscle power deficits [37], such as the five sit-to-stand test. Interestingly, lower limb muscle power could be estimated through this test, and it has been shown to be a better predictor of mortality in older adults compared to walking test velocity [38]. Notably, with advancing age, humans first stop performing power-based tasks while maintaining considerable skeletal muscle strength and mass and continuing to perform maximal strength-based tasks [2]. This can be observed, for instance, when assessing the ability to perform a jumping task in older individuals where most with advanced age are not able to jump [39]. Thus, we contend that the type of tests capable of being performed by older adults should also be considered as a criterion for classifying the degree of powerpenia, in addition to the performance of the test itself, taking into consideration the individual’s characteristics.

## **Future Research Prospects**

For an effective introduction of the powerpenia concept and leverage for a new conceptual framework, we assert that widespread acceptance from researchers who investigate this topic is needed. We anticipate that there may be some reluctance on the part of some researchers. Thus, a group opinion by a panel of experts would be required in the near future, for instance by applying the Delphi method.

**Fig. 1:**
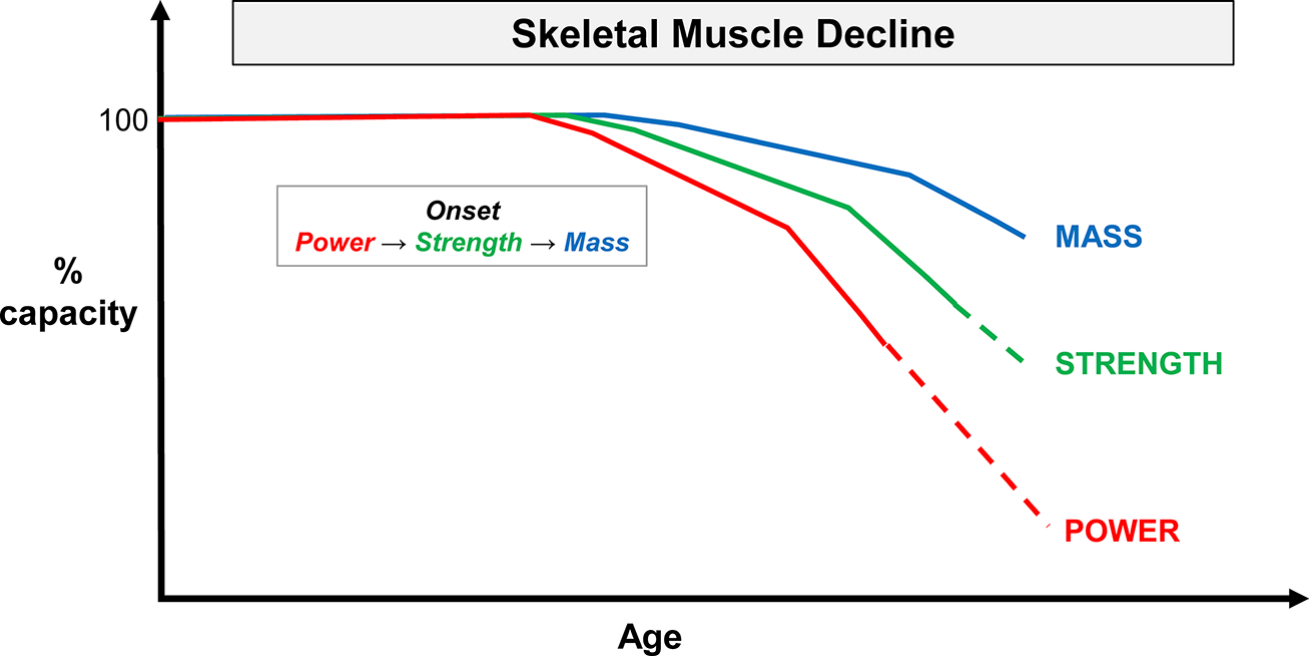
A hypothetical model for the onset and rate of skeletal muscle power, strength, and mass decline with advancing age. The dashed line means that individuals cannot perform all power or strength tasks

Another future prospect relates to the degree of relevance of skeletal muscle power, strength and mass as biomarkers of health. For instance, while several studies indicate that the rate of loss with age is higher for skeletal muscle power, followed by strength and then muscle mass [9, 13, 17], it is not clear whether the onset of decline has the same temporal order and which approach is the best to determine such onset. Considering the previous findings [9, 12, 17], we hypothesize that the onset and rate of decline of skeletal muscle with aging occurs in this order: first and higher in power, followed by maximal strength, and (ultimately) muscle mass (Fig. 1). However, as skeletal muscle atrophy with aging is muscle-specific [40], it is worth noting that differences between the onset of decline of these variables may be also muscle-dependent, meaning that there could be differences between the tonic and phasic muscles, as between the lower and upper limbs [12], although differences may exist between individuals of different sexes and ages [41, 42].

A conceptual framework and operational algorithm would also need to be designed to assess and determine meaningful powerpenia. Besides identifying the power quantification approach and which power parameter would be most appropriate, as previously mentioned, we contend that the conceptual framework should contemplate testing categories that are selected according to the individual’s age, physical status, and physical and cognitive limitations. For instance, although lower limb power is in general more relevant than that of the upper limbs, it is important to explore the importance of muscular power in the upper limbs in people who are unable to move their lower limbs, meaning powerpenia could be an inclusive concept.

The last general research prospect we propose is to further explore the relevance of powerpenia in different clinical and health contexts. As modern humans in industrialized countries continue to adopt an increasingly inactive and sedentary lifestyle, and non-communicable diseases are increasing globally [43], the ability of current and future generations to generate musculoskeletal power may be adversely affected. This is particularly relevant since the retirement age is increasing in many countries. As such, future research should investigate the impact of these conditions on powerpenia and other biomarkers through multicentric and multicultural study designs. For example, does the ability to perform power-based tasks reflect the decline in muscle health and quality of life earlier than muscle strength or muscle mass in healthy and clinical individuals? Also, how are skeletal muscle power, strength and mass affected by different diseases compared to other biomarkers? Does powerpenia affect other populations than older adults, such as children? By investigating these (and other) questions, the relevance of the powerpenia concept in different contexts could be determined.

## Conclusion

In this current opinion manuscript, we propose the introduction of the *powerpenia* concept as a biomarker of healthy aging, to specifically address the skeletal muscle power loss due to aging, clinical conditions, and/or physical inactivity. This introduction is justified by the fact that skeletal muscle power and strength decline differently with aging and disease, depend on different underlying factors, and have different influences on human physical function. Thus, we contend that the powerpenia concept should be distinguished from dynapenia (i.e., strength loss) and sarcopenia (i.e. muscle mass loss). Several research questions arise from this proposal that need to be addressed in the future, in particular the definition of the conceptual framework and operational algorithm to assess the individual’s powerpenia. Together, but with different focuses, sarcopenia, dynapenia, and powerpenia should be considered biomarkers of healthy aging.

### Electronic Supplementary Material

Below is the link to the electronic supplementary material.

Supplementary Material 1

## Data Availability

In the supplemental file.
